# Pathologic and biologic response to preoperative endocrine therapy in patients with ER-positive ductal carcinoma *in situ*

**DOI:** 10.1186/1471-2407-9-285

**Published:** 2009-08-18

**Authors:** Yunn-Yi Chen, Sandy DeVries, Joseph Anderson, Juan Lessing, Rebecca Swain, Koei Chin, Veronica Shim, Laura J Esserman, Frederic M Waldman, E Shelley Hwang

**Affiliations:** 1 Department of Pathology, University of California San Francisco, San Francisco, USA; 2 Department of Laboratory Medicine, University of California San Francisco, San Francisco, USA; 3 Department of Surgery, University of California San Francisco, San Francisco, USA; 4 Helen Diller Family Comprehensive Cancer Center, University of California, San Francisco, USA; 5 Department of Surgery, Kaiser Permanente, Oakland, USA

## Abstract

**Background:**

Endocrine therapy is commonly recommended in the adjuvant setting for patients as treatment for ductal carcinoma *in situ *(DCIS). However, it is unknown whether a neoadjuvant (preoperative) anti-estrogen approach to DCIS results in any biological change. This study was undertaken to investigate the pathologic and biomarker changes in DCIS following neoadjuvant endocrine therapy compared to a group of patients who did not undergo preoperative anti-estrogenic treatment to determine whether such treatment results in detectable histologic alterations.

**Methods:**

Patients (n = 23) diagnosed with ER-positive pure DCIS by stereotactic core biopsy were enrolled in a trial of neoadjuvant anti-estrogen therapy followed by definitive excision. Patients on hormone replacement therapy, with palpable masses, or with histologic or clinical suspicion of invasion were excluded. Premenopausal women were treated with tamoxifen and postmenopausal women were treated with letrozole. Pathologic markers of proliferation, inflammation, and apoptosis were evaluated at baseline and at three months.

Biomarker changes were compared to a cohort of patients who had not received preoperative treatment.

**Results:**

Median age of the cohort was 53 years (range 38–78); 14 were premenopausal. Following treatment, predominant morphologic changes included increased multinucleated histiocytes and degenerated cells, decreased duct extension, and prominent periductal fibrosis. Two postmenopausal patients had ADH only with no residual DCIS at excision. Postmenopausal women on letrozole had significant reduction of PR, and Ki67 as well as increase in CD68-positive cells. For premenopausal women on tamoxifen treatment, the only significant change was increase in CD68. No change in cleaved caspase 3 was found. Two patients had invasive cancer at surgery.

**Conclusion:**

Preoperative therapy for DCIS is associated with significant pathologic alterations. These changes may be clinically significant. Further work is needed to identify which women may be the best candidates for such treatment for DCIS, and whether best responders may safely avoid surgical intervention.

**Trial Registration:**

ClinicalTrials.gov NCT00290745

## Background

Ductal carcinoma in situ (DCIS) was diagnosed in over 60,000 women in the United States in 2008[1]. The incidence of DCIS has risen almost 5-fold over the last 15 years, and now represents 25–30% of all mammographically detected breast cancers[2,3]. Moreover, studies suggest that the undetected disease reservoir of DCIS could be even larger. Autopsy series in women dying of causes other than breast cancer show that over 10% of whole breast specimens may harbor DCIS not previously recognized [4-6]. Since DCIS is rarely palpable, mammography is the primary mode of detection. As mammographic screening has become more sensitive and widespread, more clinically occult preinvasive disease continues to be detected.

The current treatment of DCIS is based on a presumption that DCIS is a non-obligate precursor of invasive breast cancer. There is a paucity of natural history studies since DCIS is generally surgically resected upon diagnosis. The few retrospective reports of women who had biopsies that were assumed to be benign but on later review were found to have DCIS report a 20–50% risk of invasive cancer in the twenty years after biopsy [7-12]. Without validated measures with which to stratify future risk for invasive cancer, the current goal of all DCIS treatment is prevention of cancer progression through surgery, radiation, hormonal therapy, or a combination of these modalities[7]. In fact, despite the 99% survival rate from DCIS, these aggressive treatments are not much different than those recommended for the invasive cancers these interventions are aimed to prevent. Thus, current therapy for DCIS may represent overtreatment for many women who may never progress to invasive cancer. Nevertheless, expectant management alone is not currently considered an acceptable alternative for most women, due to fear of invasive progression.

In prospective randomized trials, adjuvant tamoxifen and aromatase inhibitor (AI) have both been associated with significantly reduced risk of contralateral invasive breast cancer [13-16]. One possible explanation for this observation is that endocrine therapy may prevent progression of in situ to invasive disease. If indeed the risk of DCIS progression could be reduced with primary medical therapy alone, some women with DCIS could potentially avoid the morbidity of surgery and radiation as well as derive benefit from contralateral risk reduction. Tamoxifen is already offered as adjuvant treatment for DCIS, based on data from a prospective placebo-controlled trial in women undergoing lumpectomy and radiation showing a benefit in DFS which favored tamoxifen[14]. Ongoing clinical trials are studying the role of AI in both the prevention and adjuvant settings for DCIS. However, the biologic impact of endocrine therapy directly on DCIS lesions themselves remains largely unexplored.

To our knowledge, this is the first report examining the biologic changes that accompany preoperative endocrine treatment of ER-positive pure DCIS. Our aim was to determine whether a 3-month course of preoperative endocrine therapy for DCIS could result in measurable histologic and immunohistochemical changes when compared to baseline assessments made on initial diagnostic core biopsy. To reduce possible systematic bias in immunohistochemistry (IHC) scoring arising from differences in tissue fixation of core biopsy versus surgical excision specimens, we also performed a matched analysis of IHC changes on core biopsy compared to surgical excision on controls who did not undergo preoperative treatment.

## Methods

### Treated Cases

Patients treated at the University of California San Francisco Comprehensive Cancer Center between 2003 and 2007 were offered participation in an investigator-initiated clinical trial studying the effect of preoperative endocrine therapy in DCIS. The study was reviewed and approved by the UCSF IRB in compliance with the Helsinki Declaration (CHR approval H10367-19435), and written informed consent from all participants was documented. Patients diagnosed with pure DCIS by stereotactic core needle biopsy of microcalcifications were recruited to a trial of 3 months of neoadjuvant endocrine therapy. Patients with current hormone replacement use, palpable masses, hormone receptor-negative DCIS, or clinical suspicion of invasion were excluded from study participation.

Premenopausal patients were treated with tamoxifen (20 mg PO QD), while postmenopausal patients were treated with letrozole (2.5 mg PO QD). Definitive excision consisting of either lumpectomy or mastectomy was planned per study protocol at the completion of three months of preoperative therapy.

### Untreated Controls

Patients undergoing surgery for DCIS at our institution between 2003 and 2006 who consented to have tissue banked for research were identified. Those with invasive cancer and those for whom diagnostic core biopsy blocks or surgical blocks were not available were excluded. Eighteen consecutive cases were evaluable for all biomarkers assessed.

### Histopathology and Biomarker Assessment

Hematoxylin and eosin (H&E)-stained sections from all pre-treatment and post-treatment biopsies were reviewed by the study pathologist (YC). Diagnosis was rendered according to the criteria established by Page et al[17] and the WHO classification system[18]. Grading was based on the evaluation of cytonuclear features and divided into three grades (1 to 3). Other features of DCIS were also recorded including architectural pattern, presence and type of necrosis, microcalcifications, associated foamy macrophages and multinucleated giant cells.

Immunohistochemistry was performed on formalin-fixed, paraffin-embedded tissue sections using the streptavidin-biotin peroxidase method. Antibodies and dilutions used were: ER (DAKO, Carpinteria, CA) at 1:400 dilution; PR (Novocastra) at 1:25; HER2 (Zymed) at 1:200; Ki-67 (DAKO) at 1:100; CD68 (DAKO) at 1:100; and cleaved caspase 3 (Cell Signaling, Beverly, MA) at 1:200.

Antigen retrieval was achieved using either heat-induced epitope retrieval in 10 mM citrate buffer at pH 6.0 (for ER, PR, CD68, and caspase 3), incubation with Ficin (Zymed) at 37°C (for HER2), or 0.01% trypsin digestion followed by heat treatment in 10 mM citrate buffer (for Ki-67). Slides were blocked in 3% H_2_O_2 _and then incubated with the primary antibodies. *HER2 *gene amplification was determined by FISH analysis using the PathVysion *HER2 *DNA Probe Kit (Vysis Inc., Downer's Grove, IL) according to manufacturer recommendations.

In order to minimize the impact of IHC staining variability in the analysis, specific care was taken to ensure that matched pretreatment and postreatment specimens were stained in the same run.

### Scoring of Biomarkers

All immunostains were evaluated by two pathologists (YC and RS) and scored randomly so that pre- and post-treatment samples were evaluated independently for both cases and controls. The scoring was perfomed without knowledge of other immunostains from the same specimen. Pathologists were also blinded to the scoring of the paired pre- or post-treatment sample.

*ER and PR *were scored by evaluating the percentage and intensity of stained tumor nuclei (H-score) as previously described[19,20]. Staining intensity ranged from 0 to 3+, with 0 representing no staining, 1+ weak staining, 2+ moderate staining, and 3+ strong staining. Percentages of positive tumor cells in each staining intensity category were recorded. Results of ER and PR were expressed as the H-score where: H-score = (1 × %1+) + (2 × %2+) + (3 × %3+).

*HER2 *was scored by criteria established by the HercepTest (DAKO), using a 0–3 scale, based on staining intensity of tumor cells. Staining intensities 0 and 1 were considered negative; intensity 2 indeterminate, and intensity 3 positive for HER2 protein overexpression. FISH analysis was performed on all cases with staining intensity 2–3 by immunohistochemistry, and cases showing a ratio of *HER2*:centromere 17 copy number greater than 2.0 were considered positive for gene amplification.

*Ki-67 *staining was used to establish a proliferation index. Slides were first scanned at low-power magnification to select DCIS foci with highest mitotic activity. Where possible, at least 500 tumor cells were counted in these mitotically active areas. Proliferation index was obtained by the percentage of tumor nuclei that were labeled by Ki-67.

*CD68 *staining was used to identify DCIS infiltrating macrophages. Macrophage density was recorded by scanning at low-power magnification to choose DCIS foci with highest concentration of CD68 positive cells. The number of intraductal CD68 positive macrophages was counted in three high-power fields (hpf, 400×). The macrophage density was expressed as the mean number of macrophages per hpf.

*Cleaved caspase 3 *staining was used to evaluate activation of apoptosis, and was interpreted as positive in tumor cells with strong granular staining in the cell. Where possible, a minimum of 500 tumor cells was scored in each sample. Only predominantly intact cells were counted. The results were expressed as the percentage of tumor cells expressing cleaved caspase 3.

### Statistics

A non-parametric rank-sum test for non-normalized distribution of data was used to compare changes in IHC markers between cases and controls, as well as between baseline and treated cases. Significance was established at a *p *value of < 0.05.

## Results

### Patient Characteristics

From 2003 to 2007, 23 patients completed the study protocol with both pre- and post-treatment materials available for analysis. Overall, the treatment was well tolerated. One patient (H-24) was discontinued from the study; she was taken off tamoxifen on day 31 due to an arterial embolic event.

Two premenopausal patients declined excision and chose to continue the endocrine treatment. However, follow-up post-treatment core needle biopsies were performed on these two patients and were compared to pre-treatment samples. The remaining patients underwent definitive surgical excision following preoperative endocrine therapy. Table 1 and Additional File 1http://waldman.ucsf.edu/Waldman.Primary.Data.html summarize the clinical characteristics of the study population.

**Table 1:** Characteristics of Study Population

		Cases	
		
	Controls	Postmenopausal	Premenopausal
N	18	9	14
			
Age at diagnosis (y)	54	61	46
			
DCIS grade			
low	2	0	1
intermediate	7	5	6
high	9	4	7
			
Type of surgery			
lumpectomy	16	8	7
mastectomy	2	1	5
none	0	0	2

### Pathologic Features

The baseline pre-treatment core biopsies showed a range of DCIS nuclear grade with 12/23 patients diagnosed with non-high grade disease (Table 1). Comparison of tumor grade between pre- and post-treatment specimens showed that the nuclear grade remained the same in 14 cases, changed to lower grade in five, and became higher grade in two cases (Table 2; Additional File 2: http://waldman.ucsf.edu/Waldman.Primary.Data.html).

**Table 2:** Comparison of histologic diagnosis at baseline and following neoadjuvant anti-estrogenic therapy

ID	Age at diagnosis	Menopausal status	Baseline diagnosis^1^	Post-treatment diagnosis
H-02	56	post	G2-3 DCIS, LCIS	G2 DCIS, G2 IDC (1.2 cm), LCIS
H-04^2^	41	pre	G2-3 DCIS	G2-3 DCIS
H-07	48	pre	G2 DCIS	G2 DCIS
H-14	65	post	G1-2 DCIS	ADH
H-15	49	pre	G2 DCIS	G1-2 DCIS, ADH
H-16^2^	60	post	G2 DCIS	G3 DCIS, G2 IDC (1.8 cm)
H-17	45	pre	G1 DCIS, ADH, FEA	G1 DCIS, ADH, FEA
H-18	53	pre	G3 DCIS	G3 DCIS
H-19	47	pre	G2 DCIS, ADH	G1 DCIS
H-20	55	post	G1-2 DCIS	G2 DCIS
H-21	52	pre	G2 DCIS	G2 DCIS
H-22	65	post	G1-2 DCIS	G1 DCIS, ADH
H-23	44	post	G2 DCIS	G1 DCIS
H-24	42	pre	G2 DCIS, FEA	G2-3 DCIS, FEA
H-27	43	pre	G2-3 DCIS	G2 DCIS
H-29	52	post	G3 DCIS	ADH
H-30^2^	78	post	G3 DCIS	G3 DCIS
H-31^2^	52	post	G2-3 DCIS	G2-3 DCIS
H-33	41	pre	G2-3 DCIS	G2-3 DCIS
H-34	42	pre	G2 DCIS	G1-2 DCIS
H-35	68	post	G3 DCIS	G3 DCIS
H-36	44	pre	G2-3 DCIS	G2-3 DCIS
H-38^2^	46	pre	G2-3 DCIS	G2-3 DCIS

^1^abbreviation: G: grade; DCIS: ductal carcinoma in situ; IDC: invasive ductal carcinoma; LCIS: lobular carcinoma in situ; ADH: atypical ductal hyperplasia; FEA: flat epithelial hyperplasia.

^2^Her2 IHC 3+

Extent of surgery was guided by mammographic extent of calcifications. In two patients, post-treatment surgical specimens demonstrated atypical ductal hyperplasia (ADH) only. These patients had residual mammographically visible calcifications following core biopsy, with calcifications confirmed pathologically on the post-treatment surgical specimen, but no DCIS was seen associated with microcalcifications following treatment.

**Figure 1:**
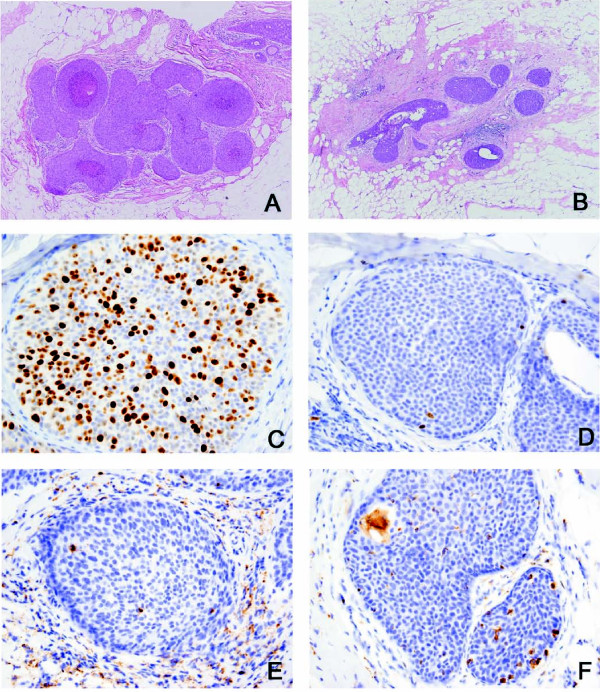
**Histology of baseline (A, C, E) and endocrine-treated (B, D, F) DCIS from patient H-20 (magnification: 100×)**. A, B: H&E stain of baseline (A) and treated (B) samples. The treated DCIS is less distended and demonstrates increased periductal sclerosis and inflammation compared to baseline. C, D: Ki67; reduction in Ki-67 after treatment compared to baseline; E, F: CD68 (inset: 400×); increased CD68-positive macrophages after treatment compared to baseline.

DCIS was identified in 21 patients following endocrine treatment. Morphologically, the post-treatment samples were less distended and demonstrated increased periductal fibrosis and inflammation when compared to the pre-treatment biopsies (Figures 1a, 1b). Treated samples also had more pronounced multinucleated histiocytes and degenerated cells within DCIS and ADH. In addition to multinucleated histiocytes and degenerated cells, the ADH in post-treatment samples demonstrated microcalcifications similar to those noted in the pre-treatment DCIS or adjacent post-treatment DCIS, suggesting some of the ADH could represent treated DCIS but with a lesser degree of cytoarchitectural features for diagnosis of DCIS. Two postmenopausal patients were also found to have intermediate grade, ER-positive invasive ductal carcinoma, measuring 1.2 and 1.8 cm respectively.

#### Biomarker Expression

The range of IHC staining for ER, Ki67, CD68, and caspase 3 for all three grades of DCIS is illustrated in Additional File 3. Changes in DCIS IHC expression from baseline to treated specimens are summarized in Table 3. The magnitude of change in treated cases and untreated controls were compared.

**Table 3:** Change in biomarkers with anti-estrogenic treatment

	Controls (n = 18)	All Cases (n = 23)		Postmenopausal Cases (n = 9)		Premenopausal Cases (n = 14)	
				
Biomarker	Mean change^1^	Mean change	*p*^2^	Mean change	*p*	Mean change	*p*
ER (H-score)^3^	-55.5 ± 22.2	-92.1 ± 23.7	0.21	-113.8 ± 26.2	0.07	-78.2 ± 35.4	0.62
PR (H-score)^3^	-38.8 ± 18.3	**-110.1 ± 18.7**	**0.03**	**-169.2 ± 18.8**	**0.001**	-72.1 ± 22.0	0.40
Ki-67^4^	-3.2 ± 1.7	**-10.2 ± 1.8**	**0.007**	**-14.4 ± 3.6**	**0.013**	**-7.5 ± 1.6**	**0.037**
CD68^4^	-0.06 ± 1.7	**34.1 ± 8.3**	**<0.0001**	**29.4 ± 5.8**	**<0.0001**	**37.2 ± 13.2**	**0.002**
caspase 3^5^	0.8 ± 0.7	0.1 ± 0.4	0.15	-0.1 ± 0.7	0.13	0.2 ± 0.5	0.33

^1^Data shows mean increase (positive values) or mean decrease (negative values) in quantitative immunohistochemical staining between diagnostic core biopsy and surgical excision. Cases were treated with neoadjuvant anti-estrogenic therapy; controls did not undergo neoadjuvant therapy.

^2^Rank-sum test, controls as referent group.

^3^H-score is weighted measure of intensity and percentage of positive cells (see Methods).

^4^Percent of cells staining positive.

^5^Number of CD68-positive macrophages per high power field within DCIS ducts.

#### ER

Hormone treatment resulted in a reduction of ER expression as demonstrated by the ER H-score. Although there was downregulation of ER expression, all DCIS lesions remained ER-positive. Reduction of ER staining was modest in the majority of the patients but at least 50% in 9 of 23 patients. This reduction was most pronounced among the postmenopausal women treated with letrozole, where it approached statistical significance.

#### PR

Endocrine treatment resulted in a marked reduction in PR expression. Three cases completely lost PR staining after treatment (H-score = 0) while all the other cases remained PR-positive. Nine of 23 cases had at least 50% downregulation in percentage of tumor cells staining positive for PR. This reduction in PR expression was significant among all cases, but stratified analysis showed that the reduction in PR was limited to the postmenopausal group treated with letrozole.

#### Ki-67

**Figure 2:**
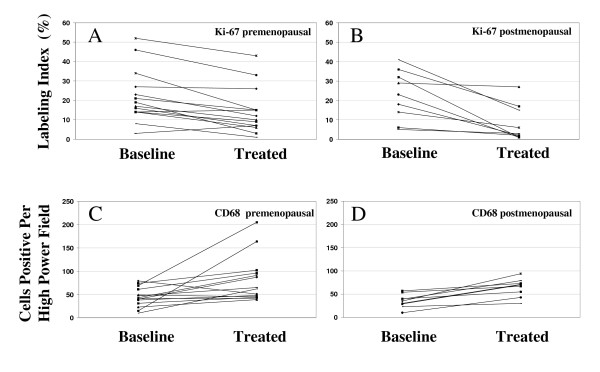
**Changes in Ki67 labeling index (A, B) and CD68-positive macrophage count (C, D) between baseline and treated DCIS**. There was significant reduction in Ki67 in premenopausal cases (p = 0.04) and in postmenopausal cases (p = 0.01). Similarly, CD68-positive macrophage density increased in both premenopausal (p = 0.002) and in postmenopausal cases (p < 0.0001). Significance was determined by the rank-sum test comparing treated to baseline values.

A significant reduction in Ki-67 labeling index was observed after endocrine therapy among both pre- and post-menopausal patients (Figures 1c, 1d). This change was significant when compared to untreated controls. DCIS nuclear grade was correlated with mean pre-treatment Ki67 (grade 1: 7.7%; grade 2: 17.4%; grade 3: 23.5%), although this finding was not statistically significant (p = 0.09). Notably, grade 3 DCIS showed the greatest mean reduction of Ki67 with treatment (13.3%), compared to grade 1 and grade 2 DCIS (6.5% and 6.1% respectively). The mean reduction in Ki-67 in postmenopausal cases was greater than that observed in premenopausal cases (Figures 2a, 2b).

#### Macrophage density

Analysis of macrophage density by CD68 showed that endocrine therapy was associated with a significant increase of macrophages within the lesional ducts compared to controls (Table 3; Figures 2c, 2d). All except two cases demonstrated an increase in macrophage density in post-treatment specimens. The change in macrophage density was correlated with the morphologic findings of many foamy macrophages and multinucleated giant cells identified in the post-treatment samples (Figures 1e, 1f).

#### HER2-neu

Of the 23 cases, five showed both *HER2 *protein overexpression (staining intensity 3 on immunohistochemistry) and/or *HER2 *gene amplification (*HER2 *gene to centromere 17 ratio > 2). Neoadjuvant endocrine therapy did not alter the HER2 status in any of the DCIS lesions. Interestingly, the reduction in Ki-67 did not differ significantly between HER2-positive and HER2-negative cases.

#### Cleaved caspase 3

There was no significant treatment effect on apoptosis as measured by the cleaved caspase 3 assay. Among those treated with endocrine therapy, twelve cases showed increased expression of cleaved caspase 3, although these changes were of small magnitude (mean change 0.1, range -6.5 to 6).

## Discussion

The aim of our study was to determine the effects of preoperative endocrine therapy on morphology and biomarker expression in DCIS. We found that administration of anti-estrogenic treatment for DCIS resulted in marked morphologic changes, decreased proliferation, and protein expression changes. Changes were compared to controls who did not undergo preoperative treatment, confirming that our findings were associated with intervening therapy rather than differences in tissue fixation between core biopsy and surgical specimens. Endocrine therapy also increased macrophage density within DCIS ducts. However, a significant change in apoptosis as measured by cleaved caspase 3 was not observed, suggesting that apoptosis was not significantly affected by this short course of treatment.

Clinical trials of neaodjuvant endocrine therapy in estrogen-positive invasive breast cancer have demonstrated a clear clinical objective response of at least 35% for both tamoxifen and aromatase inhibitors at 3 or 4 months [21-23]. If hormone therapy also induces regression in DCIS, alterations in morphology of DCIS would have been expected. In the present study, most cases treated with neoadjuvant therapy showed morphologic changes in the DCIS. These included a decrease in duct distention, an increase in periductal sclerosis and scattered degenerated cells, as well as an increase in macrophage infiltrate. These morphologic changes are compatible with treatment effect and may represent regression of DCIS. Furthermore, two of the 23 cases treated with endocrine therapy demonstrated only ADH without residual DCIS in the post-treatment excision specimens. We cannot entirely exclude the possibility that all DCIS was removed during the initial core biopsy for these two patients. However, the ADH seen in the post-treatment specimens was present immediately adjacent to biopsy site changes, demonstrated similar microcalcifications as those observed in the original DCIS, and showed similar cytologic features but less developed architecture as in the corresponding pre-treatment DCIS. Furthermore, degenerating epithelial cells and multinucleated giant cells were noted within the ADH ducts. Therefore, the morphologic features of these post-treatment ADH suggest that they may represent altered DCIS associated with treatment.

The anti-tumor effect of targeted endocrine therapy could be attributed to decreased proliferation and/or increased apoptosis at the cellular level. In this study, neoadjuvant endocrine therapy reduced cellular proliferation in DCIS cells, as measured by reduction in Ki-67. Our findings are in agreement with data reported by Boland et al. which showed a significant reduction in the percentage of proliferating cells in ER-positive DCIS following estrogen withdrawal[24]. Previous studies have also demonstrated an antiproliferative effect of tamoxifen and aromatase inhibitors in invasive breast cancer [25-31]. Most recently, in a study evaluating the DCIS component in prospective randomized window trial of letrozole versus anastrozole for ER-positive postmenopausal invasive cancer, a significant drop in Ki67 was seen for both groups in the DCIS component of the tumor[32]. In aggregate, these studies confirm that endocrine therapy effectively reduces proliferation in DCIS.

However, endocrine therapy did not have a discernible effect on apoptosis as measured by the cleaved caspase 3 assay. The lack of an observed apoptotic effect could be due to the limited sample size, or other apoptotic pathways not detected by the caspase 3 assay. Alternatively the drug concentrations at the DCIS site after oral tamoxifen or letrozole may have been below the levels required for activation of such programmed cell death pathways. Previous studies have shown conflicting results on the association between endocrine treatment and apoptosis. While in vitro[33,34], animal model[35] and one clinical[36] study demonstrated activation of apoptosis by endocrine therapy, other clinical trials on invasive breast cancer failed to show increased apoptosis by tamoxifen, raloxifene, or letrozole[31,37,38]. Further studies are required to elucidate the effect on apoptotic activity by endocrine therapy in DCIS.

Short-term treatment with AI resulted in downregulation of ER and PR expression in the DCIS cells, an effect not seen with tamoxifen treatment. This is consistent with studies in invasive breast cancer which also demonstrated changes in ER and PR expression after endocrine therapy[26,29-31,37,39]. The mechanism for ER downregulation by endocrine therapy is unclear. A previous study suggested that the effect was likely a post-transcriptional modification as there was no change of ER mRNA level[40]. On the other hand, the *PR *gene is an estrogen-regulated gene, so drugs with endocrine activity would be expected to reduce PR expression. Indeed, post-treatment PR H-score of less than 10 was noted in four cases, all of whom were treated with letrozole.

In this cohort, five cases overexpressed Her2. There was no difference in Ki-67 reduction between Her2-positive and Her2-negative cases in this small study. The IMPACT trial which compared neoadjuvant tamoxifen to anastrozole in ER-positive invasive breast cancer has suggested that when compared to HER2-negative tumors, HER2-positive lesions tended to show less antiproliferative effect following endocrine treatment[27]. This effect may not have been detectable in this small cohort. However, the neoadjuvant therapy approach demonstrated in our study may provide an ideal model for addressing the impact of HER2 overexpression on the response to hormone therapy in DCIS in larger clinical trials.

Importantly, two of the 23 patients had invasive ductal carcinoma in addition to DCIS in post-treatment excision specimens. One of the invasive carcinomas was HER2-positive, as was its associated DCIS. In both cases, the invasive tumors were hormone receptor-positive. The invasive carcinoma was likely present at initial diagnosis, but the focus missed due to sampling error. The rate of invasive cancer seen in our study is somewhat lower than reported rates of upstaging to invasive cancer (20–25%) in patients with DCIS only on core biopsy[41,42]. This may reflect a higher degree scrutiny for invasive cancer for those patients enrolling on this study, as assessed on both clinical examination and radiographic work-up. Both patients with invasive cancer underwent clinical examination, mammography, and MRI prior to study entry, none of which were suspicious for invasive cancer. In the current study, this would not have been expected to impact patient outcome as all patients had ER-positive disease and definitive surgery was performed in all but two patients who declined surgery. Thus essentially, these postmenopausal patients were treated with a standard course of neoadjuvant aromatase inhibitor therapy. However, the risk of invasive disease not detected at stereotactic core biopsy is an important consideration for future trials of non-operative treatment for preinvasive disease, and underscores the importance of patient selection in the design of preoperative clinical trials in DCIS. Current efforts, including some in our own group, are focusing on efforts to improve discrimination of invasive cancer and DCIS on MRI by optimizing both image acquisition and software analysis technology to address this important need.

Finally, it is important to note that any studies seeking to evaluate non-surgical alternatives for what is currently a surgically treated disease will encounter challenges in study design, recruitment and analysis. Since DCIS is a noninvasive condition, it presents an ideal opportunity to study those patients at low risk of cancer progression, but clinical trial designs must be take into account the risk for invasive cancer, as discussed above, as well as patient acceptance of new treatment approaches, and must remain cognizant of the difficulties inherent in prospective randomization of patients between medical and surgical treatment alternatives. Many of these barriers will be overcome with greater patient and provider education.

## Conclusion

In conclusion, preoperative hormone therapy for DCIS was feasible and well tolerated, and the effect of neoadjuvant hormone therapy in the setting of DCIS was associated with significant changes in both morphology and biomarker expression. The reduction of proliferation confirms that systemically administered drug delivery to intraductal lesions is sufficient to exert a favorable biologic effect. Whether this effect is sufficient to prevent invasive progression in the long-term is an important and compelling subject for additional research. However, our study opens the door for future trials evaluating the potential role of non-operative options for DCIS. The neoadjuvant approach described in our study provides a framework for evaluating the response of DCIS to preoperative therapies currently, including trials of agents targeting ER-negative disease. Such studies will yield important insight about the mechanisms involved in breast cancer progression and will thus inform the design of more targeted prevention studies. Although preliminary, our findings can initiate the dialogue for consideration of primary hormone therapy alone for DCIS in a select population of patients. As a follow-up to this current study, we are planning a multi-center clinical trial comparing longer intervals of letrozole treatment in postmenopausal women with ER-positive DCIS to further explore the acceptability, safety and efficacy of this approach.

## List of Abbreviations

DCIS: Ductal carcinoma *in situ*; DFS: Disease-free survival; IHC: Immunohistochemistry; IRB: Institutional Review Board; H&E: Hematoxylin and eosin; FISH: Fluorescent in-situ hybridization; ADH: Atypical ductal hyperplasia.

## Competing interests

YYC, SD, JA, JL, RS, KC, VS, LJE, and FMW have no competing interests to declare. ESH – research grant from Novartis Corporation.

## Authors' contributions

YYC, SD, JA, RS, FMW: IHC staining and scoring; YYC, SD, JA, JL, RS, KC, VS: data analysis and interpretation; YYC, FMW, LJE, ESH: study conception and design; FMW, ESH: data analysis; JL, VS, LJE, ESH: execution of clinical study; YYC, SD, FMW, ESH: manuscript drafting and preparation. All authors have read and approved the final manuscript.

## Pre-publication history

The pre-publication history for this paper can be accessed here:

http://www.biomedcentral.com/1471-2407/9/285/prepub

## Supplementary Material

Additional file 1 **Clinical parameters of study population**. Clinical characteristics of study population. Click here for file

Additional file 2 **Immunohistochemistry**. Comparison of pathologic features between pre- and post-treatment specimens. Click here for file

Additional file 3 **Representative low-power figures of ER, Ki67, CD68, and caspase 3 staining in low, intermediate, and high grade DCIS**. All cases shown are post-treatment specimens. (A) ER strong staining (90%) in low grade DCIS. (B) ER weak staining (25%) in intermediate grade DCIS. (C) Ki67 strong staining (26%) in high grade DCIS. (D) Ki67 weak staining (7%) in intermediate grade DCIS. (E) CD68 strong staining (score 164) in intermediate grade DCIS. (F) CD68 weak staining (score 96) in intermediate grade DCIS. (G) caspase 3 strong staining (8.8%) in high grade DCIS. (H) caspase 3 weak staining (1.6%) in intermediate grade DCIS. Click here for file
