# Supplementary material for: Pathologic and biologic response to preoperative endocrine therapy in patients with ER-positive ductal carcinoma in situ
Source: BMC Cancer. 2009 Aug 18;9:285. doi: 10.1186/1471-2407-9-285 (PMC2744704; doi:10.1186/1471-2407-9-285)
Supplement: Additional file 1 — Clinical parameters of study population. Clinical characteristics of study population. [file 1471-2407-9-285-S1.doc]

| **Supplemental Table 1. Clinical parameters** | | | | | |  |  |  |  |
| --- | --- | --- | --- | --- | --- | --- | --- | --- | --- |
|  |  |  |  |  |  |  |  |  |  |
| **Sample ID** | **Age** | **Surgery^1^** | **# of pos nodes** | **# of total nodes** | **# of re-excisions** | **Baseline highest nuclear grade** | **Treated highest nuclear grade** | **IDC present^2^** | **IDC size (cm)** |
| H-02 | 55 | 1 | 1 | 26 | 0 | 3 | 2 | 1 | 1.2 |
| H-04 | 41 | 2 | 0 | 1 | 0 | 3 | 3 | 0 | . |
| H-07 | 48 | 2 | 0 | 2 | 0 | 2 | 2 | 0 | . |
| H-14 | 65 | 1 | 0 | 0 | 0 | 1 | . | 0 | . |
| H-15 | 48 | 3 | . | . | . | . | . | . | . |
| H-16 | 60 | 1 | 0 | 4 | 0 | 2 | 3 | 1 | 1.8 |
| H-17 | 45 | 1 | 0 | 0 | 0 | 1 | 1 | 0 | . |
| H-18 | 53 | 2 | 0 | 1 | 0 | 3 | 3 | 0 | . |
| H-19 | 47 | 3 | . | . | . | . | . | . | . |
| H-20 | 55 | 1 | 0 | 0 | 0 | 3 | 2 | 0 | . |
| H-21 | 51 | 1 | 0 | 0 | 0 | 2 | 1 | 0 | . |
| H-22 | 65 | 1 | 0 | 0 | 0 | 2 | 2 | 0 | . |
| H-23 | 44 | 1 | 0 | 0 | 1 | 2 | 2 | 0 | . |
| H-24 | 42 | 2 | 0 | 3 | 0 | 2 | 3 | 0 | . |
| H-27 | 50 | 1 | 0 | 0 | 0 | 3 | 2 | 0 | . |
| H-29 | 52 | 1 | 0 | 0 | 0 | 3 | . | 0 | . |
| H-30 | 78 | 2 | 0 | 1 | 0 | 3 | 3 | 0 | . |
| H-31 | 52 | 1 | 0 | 0 | 0 | 3 | 3 | 0 | . |
| H-33 | 41 | 1 | 0 | 0 | 0 | 3 | 3 | 0 | . |
| H-34 | 42 | 1 | 0 | 1 | 2 | 2 | 1 | 0 | . |
| H-35 | 68 | 1 | 0 | 0 | 0 | 3 | 3 | 0 | . |
| H-36 | 44 | 1 | 0 | 0 |  | 2 |  |  |  |
| H-38 | 46 | 2 | 0 | 1 | 3 | 3 | 2 | 0 | . |
|  |  |  |  |  |  |  |  |  |  |
| Coding: |  |  |  |  |  |  |  |  |  |
| 1: | Surgery type: 1=lumpectomy; 2=mastectomy; 3=no surgery | | | | |  |  |  |  |
| 2: | IDC: invasive ductal carcinoma absent (0) or present (1) | | | | |  |  |  |  |
