# Supplementary material for: Pathologic and biologic response to preoperative endocrine therapy in patients with ER-positive ductal carcinoma in situ
Source: BMC Cancer. 2009 Aug 18;9:285. doi: 10.1186/1471-2407-9-285 (PMC2744704; doi:10.1186/1471-2407-9-285)
Supplement: Additional file 2 — Immunohistochemistry. Comparison of pathologic features between pre- and post-treatment specimens. [file 1471-2407-9-285-S2.doc]

| **Supplemental Table 2: Immunohistochemistry** | | | | | | |  |  |  |  |  |  |
| --- | --- | --- | --- | --- | --- | --- | --- | --- | --- | --- | --- | --- |
|  |  |  |  |  |  |  |  |  |  |  |  |  |
| **Case/ Control^1^** | **Sample ID** | **age** | **Baseline ER H^2^** | **Treated ER H** | **Baseline PR H** | **Treated PR H** | **Baseline ki-67 %** | **Treated ki-67 %** | **Baseline CD68 per hpf^3^** | **Treated CD68 per hpf** | **Baseline caspase3 %** | **Treated caspase3 %** |
| 0 | C-01 | 49 | 290 | 0 | 260 | 130 | 2 | 3 | 16 | 20 |  |  |
| 0 | C-03 | 55 | 270 | 210 | 300 | 270 | 5 | 4 | 20 | 24 | 0.2 | 1.0 |
| 0 | C-04 | 60 | 260 | 220 | 200 | 210 | 31 | 8 | 42 | 32 | 2.4 | 2.2 |
| 0 | C-05 | 63 | 300 | 300 | 210 | 170 | 12 | 10 | 20 | 19 |  | 1.8 |
| 0 | C-06 | 49 | 80 | 70 | 210 | 240 | 17 | 18 | 39 | 24 | 4.6 | 10.2 |
| 0 | C-07 | 55 | 270 | 300 | 260 | 120 | 4 | 3 | 14 | 13 |  |  |
| 0 | C-08 | 39 | 220 | 80 | 260 | 240 | 9 | 4 | 25 | 34 | 2.0 | 2.2 |
| 0 | C-09 | 47 | 240 | 210 | 260 | 210 | 44 | 41 | 39 | 35 | 0.4 | 2.0 |
| 0 | C-10 | 54 | 290 | 280 | 300 | 250 | 9 | 10 | 28 | 30 | 1.6 | 1.2 |
| 0 | C-11 | 59 | 270 | 200 |  |  |  |  |  |  | 1.7 | 8.2 |
| 0 | C-13 | 43 | 130 | 230 | 90 | 220 | 6 | 19 |  |  |  | 5.2 |
| 0 | C-14 | 54 | 250 | 60 | 190 | 50 | 36 | 29 | 10 | 9 | 5.0 | 5.8 |
| 0 | C-15 | 70 | 300 | 300 | 270 | 230 | 12 | 6 | 43 | 40 | 0.4 | 0.4 |
| 0 | C-16 | 60 | 290 | 300 | 260 | 240 | 27 | 17 | 12 | 14 | 3.2 | 2.4 |
| 0 | C-17 | 61 | 270 | 130 | 230 | 50 | 29 | 26 | 12 | 10 | 7.2 | 1.2 |
| 0 | C-24 | 38 | 270 | 120 | 270 | 300 | 12 | 6 | 21 | 36 | 1.2 | 0.8 |
| 0 | C-26 | 71 | 260 | 290 | 280 | 260 | 11 | 7 | 37 | 34 | 2.0 | 4.4 |
| 0 | C-27 | 52 | 300 | 260 | 300 | 300 | 4 | 3 | 4 | 7 | 0.4 | 2.0 |
| 1 | H-02 | 55 | 300 | 240 | 210 | 100 | 18 | 2 | 30 | 71 | 0.6 | 1.0 |
| 1 | H-04 | 41 | 210 | 50 | 240 | 120 | 27 | 26 | 40 | 87 | 3.2 | 0.8 |
| 1 | H-07 | 48 | 270 | 15 | 270 | 210 | 14 | 15 | 61 | 96 | 1.0 | 3.0 |
| 1 | H-14 | 65 | 270 | 270 | 180 | 120 | 6 | 2 | 57 | 73 | 0.8 | 0.2 |
| 1 | H-15 | 48 | 70 | 270 | 240 | 270 | 14 | 6 | 23 | 39 | 1.2 | 2.2 |
| 1 | H-16 | 60 | 210 | 120 | 140 | 2 | 29 | 27 | 29 | 70 | 6.8 | 2.8 |
| 1 | H-17 | 45 | 240 | 270 | 240 | 150 | 14 | 9 | 31 | 44 | 0.0 | 0.6 |
| 1 | H-18 | 53 | 270 | 10 | 240 | 220 | 52 | 43 | 43 | 91 | 4.8 | 4.2 |
| 1 | H-19 | 47 | 240 | 210 | 270 | 10 | 46 | 33 | 38 | 48 | 1.6 | 1.8 |
| 1 | H-20 | 55 | 270 | 80 | 160 | 0 | 32 | 1 | 54 | 67 | 1.2 | 0.0 |
| 1 | H-21 | 51 | 270 | 50 | 270 | 140 | 16 | 7 | 15 | 164 | 2.0 | 1.0 |
| 1 | H-22 | 65 | 240 | 140 | 210 | 80 | 14 | 6 | 35 | 94 | 2.0 | 3.2 |
| 1 | H-23 | 44 | 270 | 270 | 240 | 140 | 8 | 1 | 10 | 62 | 1.6 | 1.4 |
| 1 | H-24 | 42 | 270 | 270 | 270 | 240 | 3 | 7 | 47 | 65 | 0.6 | 2.2 |
| 1 | H-27 | 50 | 270 | 50 | 180 | 10 | 23 | 12 | 79 | 51 | 2.2 | 1.4 |
| 1 | H-29 | 52 | 270 | 160 | 240 | 10 | 23 | 1 | 10 | 43 | 2.0 | 1.6 |
| 1 | H-30 | 78 | 270 | 120 | 210 | 5 | 36 | 17 | 40 | 55 | 3.2 | 2.3 |
| 1 | H-31 | 52 | 270 | 5 | 200 | 0 | 41 | 15 | 23 | 30 | 4.4 | 9.0 |
| 1 | H-33 | 41 | 270 | 270 | 140 | 120 | 21 | 15 | 74 | 102 | 1.0 | 6.0 |
| 1 | H-34 | 42 | 80 | 20 | 160 | 180 | 17 | 10 | 49 | 45 | 1.8 | 1.0 |
| 1 | H-35 | 68 | 300 | 240 | 300 | 10 | 5 | 3 | 39 | 79 | 2.0 | 1.6 |
| 1 | H-36 | 44 | 300 | 300 | 240 | 270 | 19 | 3 | 69 | 205 | 4.6 | 2.2 |
| 1 | H-38 | 46 | 240 | 120 | 230 | 140 | 34 | 15 | 41 | 42 | 1.4 | 2.8 |
|  |  |  |  |  |  |  |  |  |  |  |  |  |
| Coding: |  |  |  |  |  |  |  |  |  |  |  |  |
| 1 | control: 0; case: 1 | | |  |  |  |  |  |  |  |  |  |
| 2 | H Score: weighted measure of intensity and percentage of positive cells (see Materials and Methods) | | | | | | | | |  |  |  |
| 3 | hpf: high power field | | |  |  |  |  |  |  |  |  |  |
