# Supplementary figures and images for: Pathologic and biologic response to preoperative endocrine therapy in patients with ER-positive ductal carcinoma in situ
Source: BMC Cancer. 2009 Aug 18;9:285. doi: 10.1186/1471-2407-9-285 (PMC2744704; doi:10.1186/1471-2407-9-285)

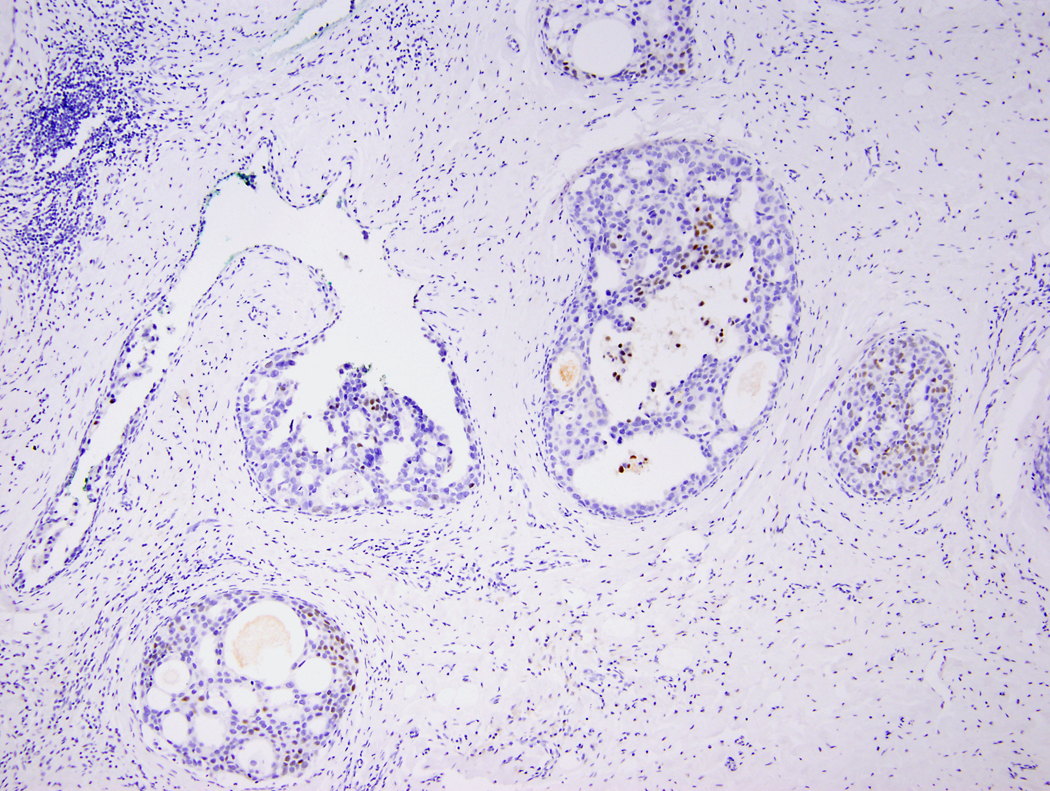

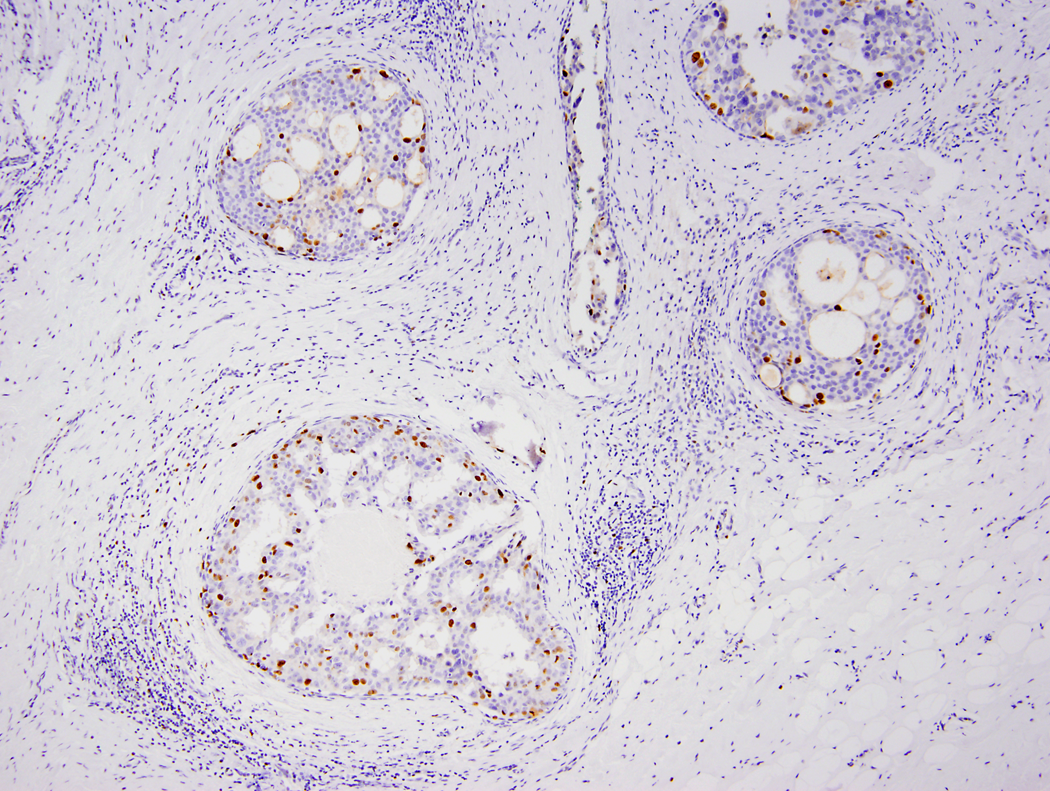

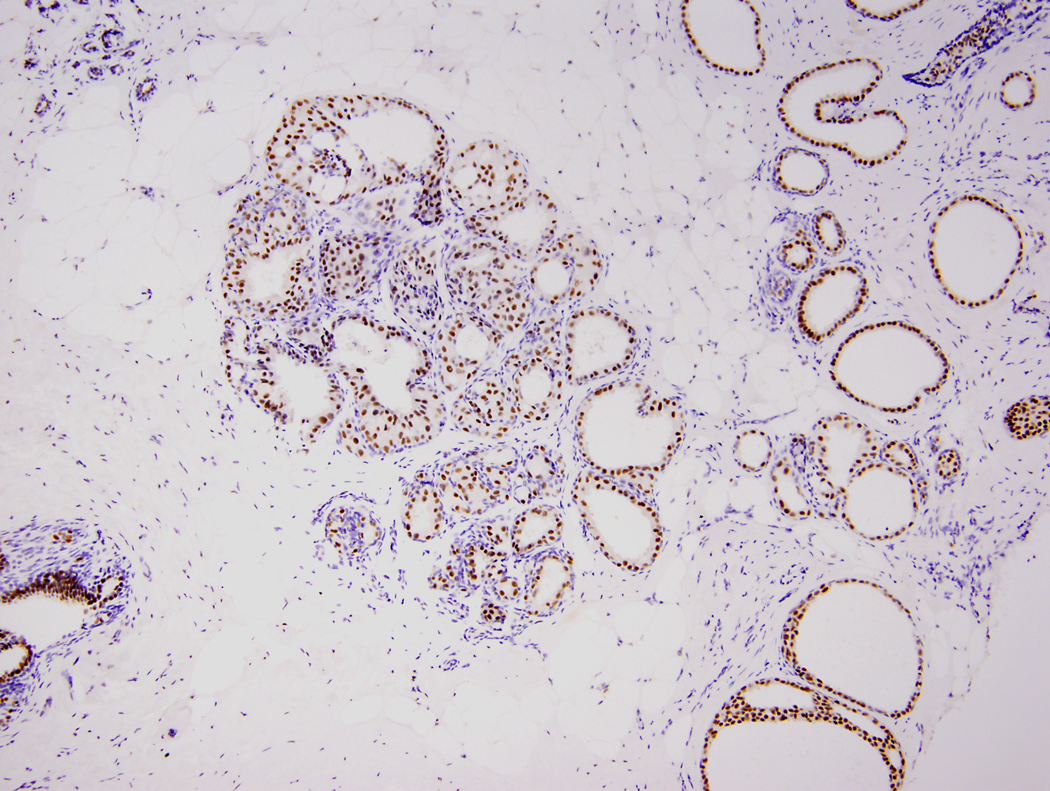

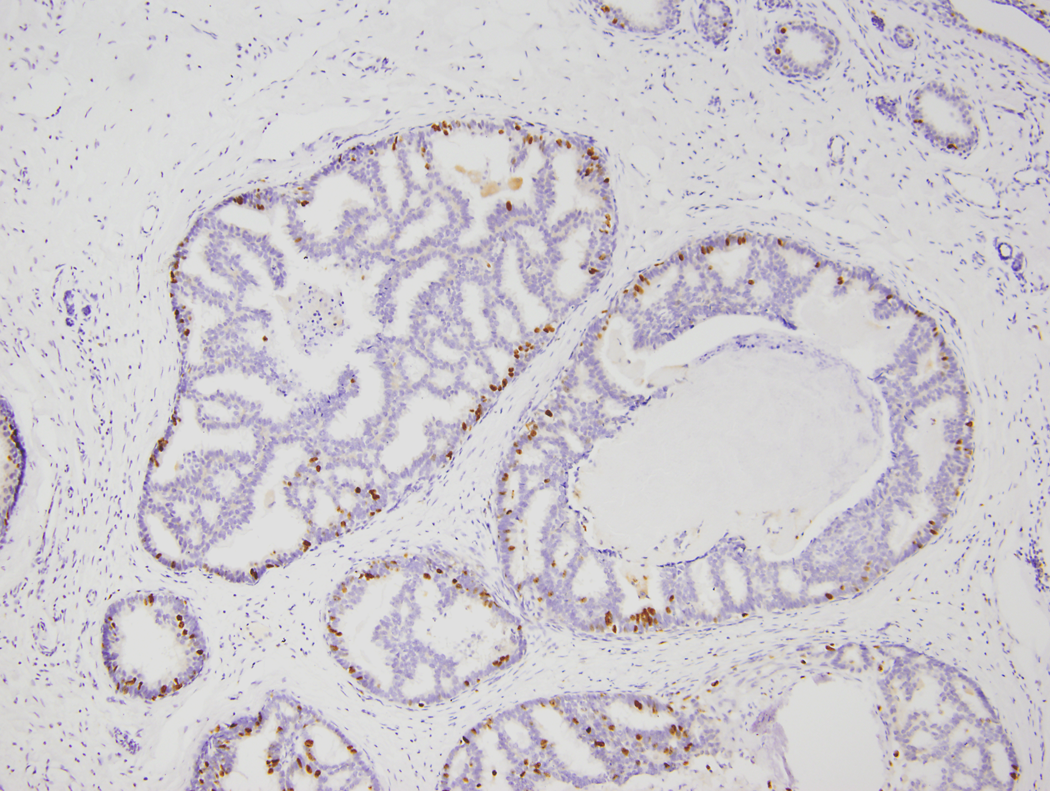

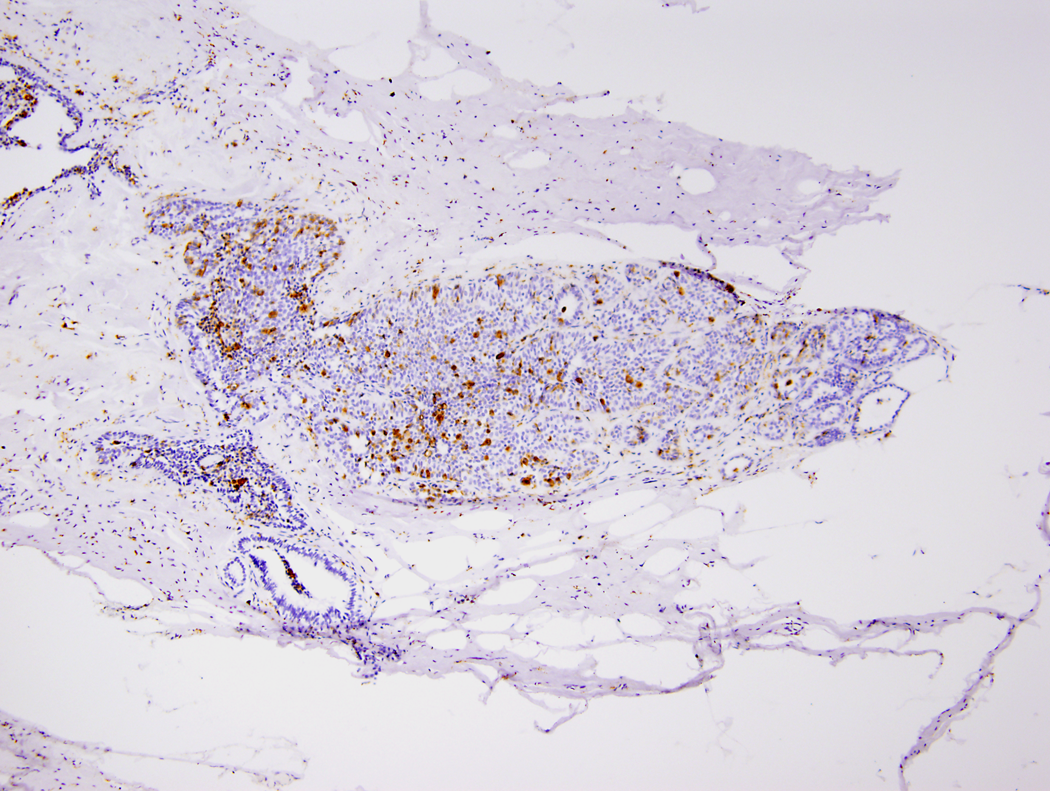

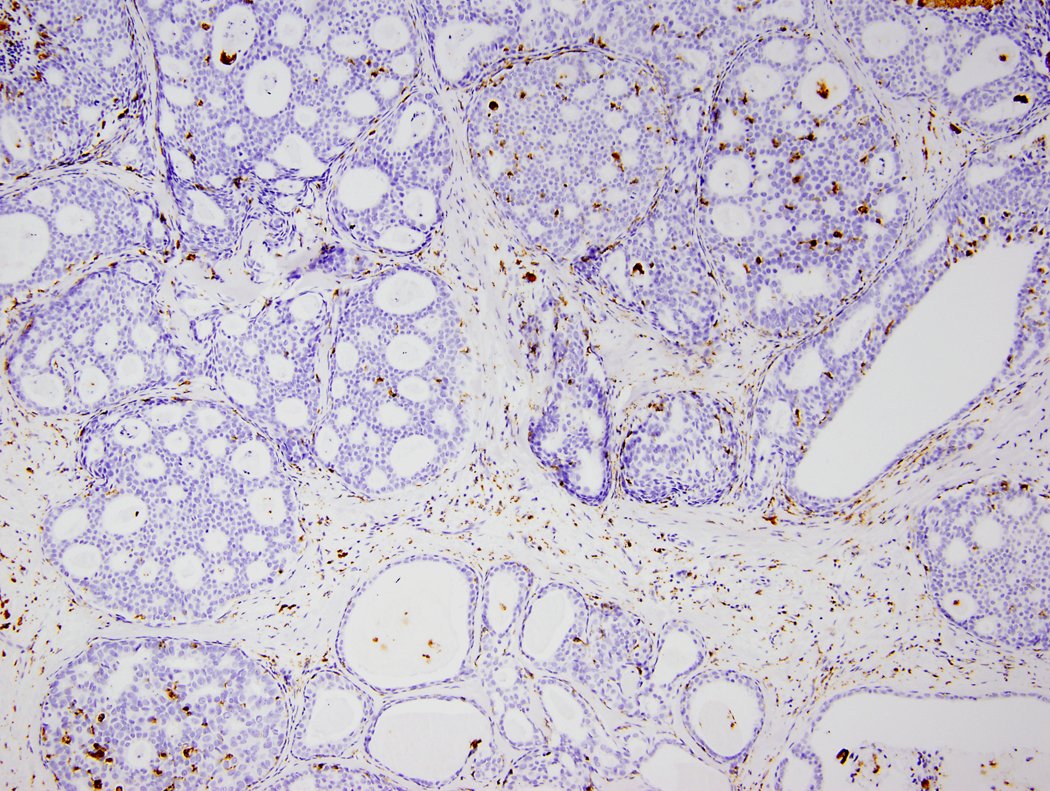

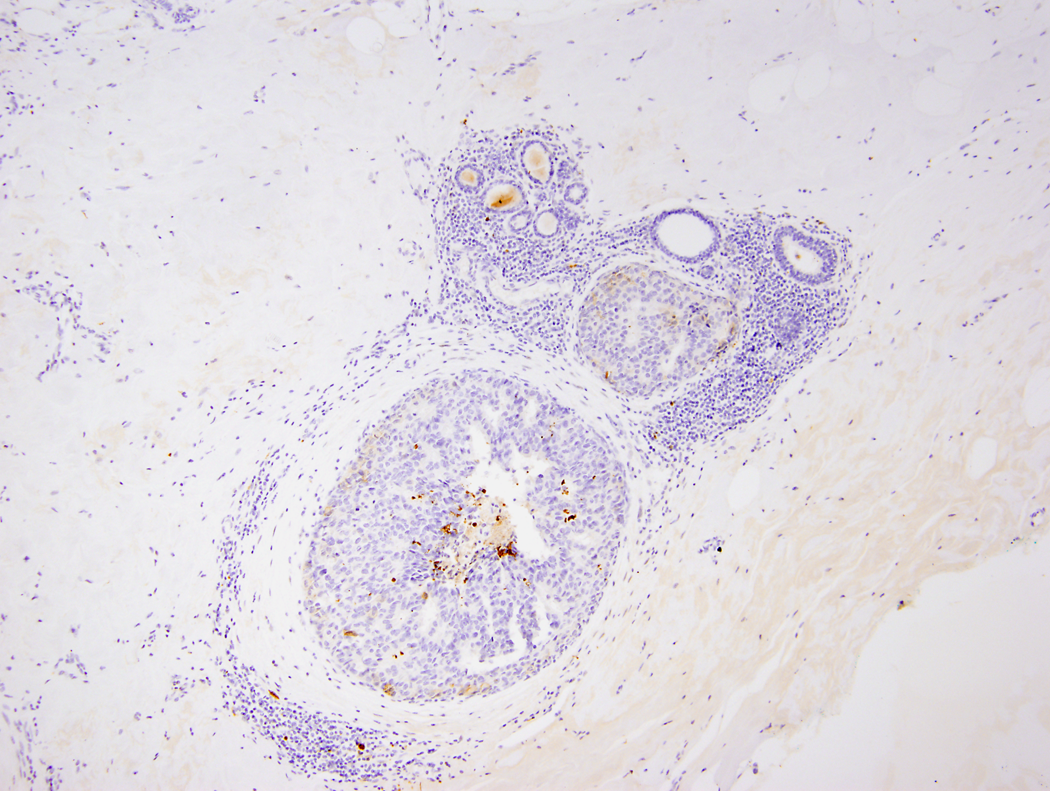

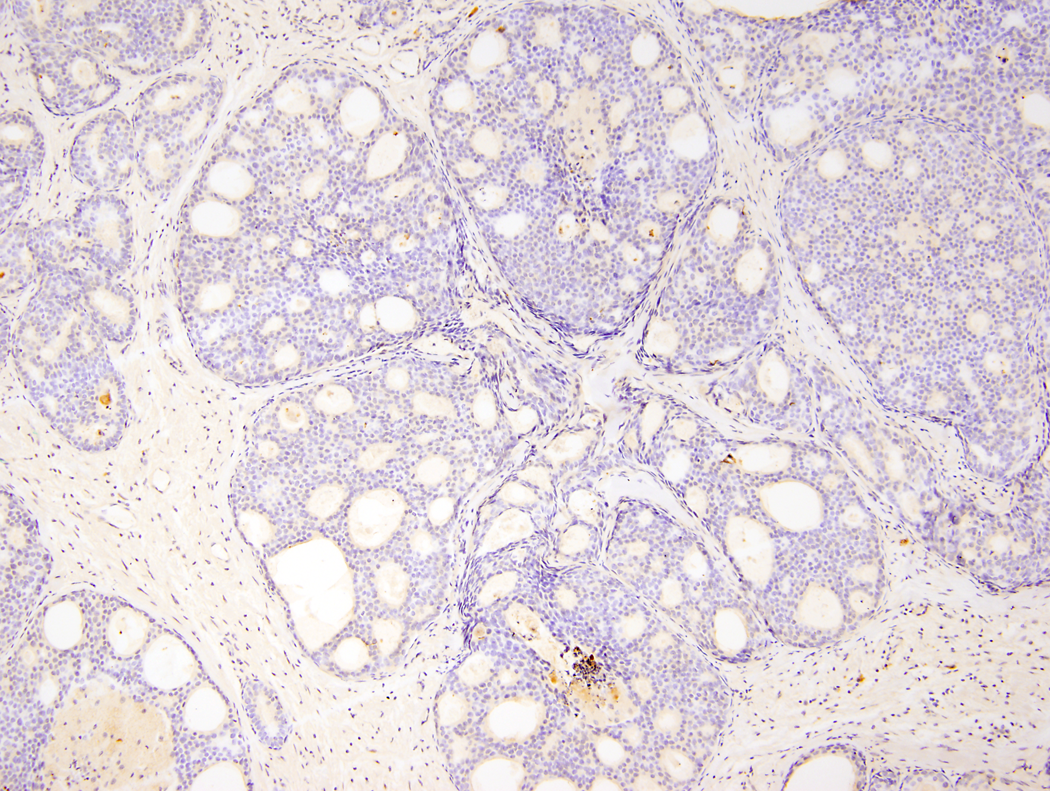


**A**

**B**

**C**

**D**

**E**

**F**

**G**

**H**

Supplement: Additional file 3 — Representative low-power figures of ER, Ki67, CD68, and caspase 3 staining in low, intermediate, and high grade DCIS. All cases shown are post-treatment specimens. (A) ER strong staining (90%) in low grade DCIS. (B) ER weak staining (25%) in intermediate grade DCIS. (C) Ki67 strong staining (26%) in high grade DCIS. (D) Ki67 weak staining (7%) in intermediate grade DCIS. (E) CD68 strong staining (score 164) in intermediate grade DCIS. (F) CD68 weak staining (score 96) in intermediate grade DCIS. (G) caspase 3 strong staining (8.8%) in high grade DCIS. (H) caspase 3 weak staining (1.6%) in intermediate grade DCIS. [file 1471-2407-9-285-S3.doc]
